# Inequality in healthcare costs between residing and non-residing patients: evidence from Vietnam

**DOI:** 10.1186/s12939-017-0581-3

**Published:** 2017-05-12

**Authors:** Hieu M. Nguyen

**Affiliations:** Department of Economics, University of Pittsburgh, 4522 Posvar Hall, Pittsburgh, PA 15260 USA

**Keywords:** Residency status, Healthcare costs, Bribery, Vietnam

## Abstract

**Background:**

Place of residence has been shown to impact health. To date, however, previous studies have only focused on the variability in health outcomes and healthcare costs between urban and rural patients. This study takes a different approach and investigates cost inequality facing non-residing patients – patients who do not reside in the regions in which the hospitals are located. Understanding the sources for this inequality is important, as they are directly related to healthcare accessibility in developing countries.

**Methods:**

The causal impact of residency status on individual healthcare spending is documented with a quasi-experimental design. The propensity score matching method is applied to a unique patient-level dataset (*n* = 900) collected at public general and specialist hospitals across North Vietnam.

**Results:**

Propensity score matching shows that Vietnamese patients who do not reside in the regions in which the hospitals are located are expected to pay about 15 million Vietnamese dongs (approximately 750 USD) more than those who do, a sizable gap, given the distribution of total healthcare costs for the overall sample. This estimate is robust to alternative matching specifications. The obtained discrepancy is empirically attributable to the differences in three potential contributors, namely spending on accompanying relatives, “courtesy funds,” and days of hospitalization.

**Conclusions:**

The present study finds that there is significant inequality in healthcare spending between residing and non-residing patients at Vietnamese hospitals and that this discrepancy can be partially explained by both institutional and non-institutional factors. These factors signal practical channels through which policymakers can improve healthcare accessibility.

## Background

Healthcare costs are an important consideration in the design of public health policies. The costs of health services affect healthcare accessibility both directly, by placing financial burdens on individual service users, and indirectly, via the magnitude of healthcare spending in relation to expenditures on other public endeavors. Within a neoclassical general equilibrium framework, agents act optimally to achieve their maximum utility. It is thus reasonable to predict that health spending is factored in this utility-maximizing mechanism in individuals' healthcare choices. Getting an insight into this process is important both to design and to evaluate healthcare programs.

Most studies in the cost literature that explore the link between health-related expenditures and health outcomes are executed at aggregate levels [1–3]. Evidence on patient-level associations is lacking. In addition, given the overall role that healthcare cost considerations play, evidence on the relative impact of each specific determinant of healthcare costs is surprisingly scanty. Part of the practical challenge lies in the economic and social costs associated with randomized control trials. From a methodological perspective, healthcare researchers are often confronted with a major difficulty in estimating counterfactual outcomes based on observational data. To be specific, the likely existence of self-selection and omitted variable biases will cause the standard exogeneity assumptions in the ordinary least squares framework to be violated. Therefore, while previous studies help conveniently identify the most likely contributors to healthcare costs, they fail to support a causal argument.

Most studies surveyed in [1] identify income as a crucial determinant of healthcare costs. Apart from income, other potential socioeconomic determinants of healthcare costs have also been investigated by previous researchers, including patients’ levels of illness, levels of education, and so forth. Ataguba et al. [4], in particular, find that there exist socio-economic gradients in self-reported ill-health in South Africa and that the burden of the major categories of ill-health and disability is greater among lower than higher socioeconomic groups.

Previous studies have also shown that much of the variation in healthcare expenditures is attributed to differences in health status, while income elasticities are negligible [5–7]. However, usage of dentistry, counselling, plastic surgery, among other largely uninsured services, still has significantly positive income elasticities [8, 9].

A largely unaddressed factor in the previous literature is the residency statuses of individual patients. Previous studies have explored residency status in direct relation to health outcomes. For example, using patient-level data from a large Canadian province, Lee et al. [10] find that place of residence does appear to influence health outcomes in patients with diffuse large B-cell lymphoma. On the other hand, Bikdeli et al. [11] report that the influence of neighborhood-specific characteristics on health outcomes, including mortality, needs to be reassessed. There has been no work that deals explicitly with the causal impact of residency status on healthcare spending using Vietnamese patient-level data.

The present study therefore aims to address these gaps in the existing literature and seeks to answer the question “To what extent is individual healthcare spending driven by residency status?” In this study, “residency” is defined as living in the province in which a health facility is situated. Place of residence is expected to have a differential effect on healthcare costs. However, previous researchers have focused exclusively on the distinction between urban and rural residents. This study follows a different direction and investigates cost inequality facing non-residing patients in a developing country.

Vietnam is an interesting case study, since the country’s health system features coexisting elements of traditional and modern medicine. While basic insurance is expected, most Vietnamese patients have to incur the costs of medical services by themselves. While most Vietnamese hospitals are publicly funded and operate under a centralized system, conforming with the Ministry of Health’s standards, the quality varies strikingly, encouraging the movement of patients from one region to another for treatment purposes. This variation naturally motivates the question posed above.

Since one of the ultimate goals of public health policies is to improve accessibility to healthcare services, the research problem presented here is especially policy-relevant, as it would signal to public health policy designers practical ways to improve hospital services and in turn the overall experiences of registered patients. It also provides significant insights into the evolution of healthcare spending trends, particularly in developing countries, and how these trends relate to health outcomes. As residency assignment is not random, a quasi-experimental design is employed to estimate the causal impact of residency status on healthcare spending that allows addressing concerns regarding endogenous regressors and omitted variable biases often raised in previous research.

## Methods

The methodological framework follows Rosenbaum & Rubin [12] and employs the propensity score matching technique proposed in their seminal paper on non-experimental causal inferences. The principal idea of this method is to estimate the treatment effect by comparing the treatment group and the control group based on a single score that measures the probability of receiving the treatment. To be specific, the “propensity score” matching algorithm pairs each treated unit with at least one “similar” control unit based on the estimated propensity scores. With the important results proven in [12], it is possible to estimate the treatment effect following a three-step algorithm:

- Step 1. Estimate the propensity scores
- Step 2. Choose an appropriate matching algorithm
- Step 3. Estimate the treatment effect from the predicted propensity scores

It is worth pointing out that propensity score matching does not fully eliminate the bias stemming from unobserved heterogeneity between the treatment and control groups. It is, however, more advantageous than other non-experimental methods in that the propensity score matching estimator is less prone to misspecification biases, as the outcome equation can assume a flexible functional form.

## Data

The data for this study is excerpted from Vuong & Nguyen [13]. Original data were collected between August 2014 and May 2015. Surveys were distributed to patients treated at polyclinic and specialist hospitals in Hanoi and some other provinces in the north of Vietnam. Vuong & Nguyen’s experiment concentrates on observations information demand, data sufficiency and efficiency in Vietnamese patients’ choice of health care provider. To make the sample as representative as possible, Vuong & Nguyen conducted surveys at approximately 150 hospitals across the capital Hanoi as well as the Red-river delta region. The supplementary file accompanying [13] lists all participating hospitals. Included in the survey are patients from the most popular hospitals in Vietnam, such as Bach Mai and Viet Duc, as well as general and specialist hospitals across North Vietnam. This enlarged database, consisting of 900 observations, records multiple patient socioeconomic and demographic characteristics, including financial issues, illness, insurance, healthcare costs, length of stay, and so forth.

The outcome variable of interest is *Total healthcare spending*, measured in Vietnamese dongs (VND), which represents the total monetary amount that a patient spent during his or her stay at one of the hospitals involved in the study. This composite measure consists of three major components, representing the percentages of funds used for the purpose of main treatments, for covering costs of accompanying relatives, and for “courtesy money,” given to the doctors/staff at the hospitals in exchange for preferential treatment (A detailed discussion of these components is presented below). At the time of the survey, the official exchange rate was 1 million VND = 47.2 USD.

The treatment variable is *Residency status*, a binary indicator that takes on value of 1 if a patient resides in the region where the hospital is located and 0 otherwise. While this might seem a simplistic measure of residency status, it is indeed a novel and improved measure. Previous studies focus only on the division between urban and rural residency, which effectively would fail to take into account urban/rural residents moving to urban/rural hospitals in other regions for treatment purposes. It is also worth pointing out that the focus of this paper is on the relationship between total healthcare costs and patients’ statuses of residency. A study of the specific breakdown of this relationship is perhaps better suited for future research. Future studies could, for instance, study the underlying psychological and economic reasons why non-residing patients chose to be treated at the incumbent hospitals. Finally, the impact of the treatment on the outcome is controlled for with a set of variables whose descriptions and selection processes are presented in the section that follows.

## Results

### Propensity score estimation

The probability of residing in a region where the hospital is located is estimated with a binary logistic regression:

$$ P\left({T}_i=1\Big|{X}_i={x}_i\right)=\frac{e^{X_i{\beta}_0}}{1+{e}^{X_i{\beta}_0}} $$

where *T*_*i*_ is the treatment indicator and *X*_*i*_ is a vector of observed covariates.

In order to estimate the propensity scores, I incorporate into X the factors that are commonly presumed to be associated with patients’ healthcare costs, such as insurance status, illness status, and income. While the bureaucracy associated with the medical insurance systems, especially those in developing countries like Vietnam, might influence the length and size of reimbursement for some, insurance coverage is expected to cover part of the treatment costs and thus alleviate the total spending for a large group of patients. It is also understandable that the more serious an illness is, the longer it would take for the patients to stay hospitalized. This in turn would intensify the burden on non-resident patients. Also included in the set of control variables are patients’ gender, age, and level of education. These factors are expected to influence both patient healthcare costs and residence decisions. Finally, hospitals where the surveyed patients received treatment are also incorporated. Reports on the conditions at Vietnamese hospitals identify overcrowding as a major issue at both public general and specialist hospitals in Vietnam [14, 15]. Aside from hygienic issues and efficiency problems, this overcrowding potentially encourages rationing behavior, particularly among high-income patient groups, furthering constraints on healthcare cost pressures.

One valid concern could be raised regarding the relative arbitrariness in variable selection. However, it should be noted, following [16] and [17], that including irrelevant variables has little effect on propensity score estimation, while omitting important confounders is very costly, as the results would be seriously biased. The full set of covariates used to control for the characteristic differences between the treatment group and the control group is described in Table 1.

**Table 1:** Descriptive statistics for the pooled sample

Variable	Obs	Mean	Std. Dev.	Min	Max
Residency status	900	0.54	0.4986746	0	1
Total spent	900	28.67533	43.83651	0.1	665
Gender	899	0.5828699	0.4933592	0	1
Age	899	45.13237	17.62531	1	92
Insurance status	900	0.6722222	0.4696643	0	1
Education	900	2.056667	0.5739138	1	4
Illness	900	3.027778	0.7400099	1	4
Income	900	41.84936	41.14481	0	550

*Gender* is a binary indicator that takes the value of 1 for Male and 0 for Female. *Age* is the patient’s age, measured in years. *Insurance status* denotes whether the patient is covered by insurance or not at the time of the survey. *Education* is a categorical variable that takes the values of 1, 2, 3, or 4 if the patient’s highest level of education completed is Junior High School, High School, University, or Graduate, respectively. *Illness* measures the severity of the patient’s illness on four levels 1, 2, 3, and 4, corresponding to Light, Ill, Bad, and Emergency. *Income* is the patient’s annual income, measured in Vietnamese dongs.

At first glance, it can be seen that the relative share of residing and non-residing patients in the pooled sample is similar. Patients treated at the hospitals included in the study paid on average 28 million VND in total expenditures. This is a sizable amount, compared to Vietnam’s 2015 per-capita GDP of around $2000 USD or roughly 45 million VND [18]. Of the full sample, nearly two thirds of the patients are covered by insurance, and most have severe illnesses. The highest level of education for the pooled sample is expected to be High School.

Table 2 breaks the sample down into two categories based on residency status and reports the descriptive statistics for the control group and the treatment group:

**Table 2:** Descriptive statistics within the control group and the treatment group

Variable	Obs	Mean	Std. Dev.	Min	Max
Control group					
Total spent	414	43.72645	45.48343	3	425
Gender	414	0.6231884	0.4851734	0	1
Age	414	38.2971	17.65985	1	86
Insurance status	414	0.4855072	0.5003946	0	1
Education	414	2.091787	0.5664384	1	4
Illness	414	3.147343	0.7623274	1	4
Income	414	36.00725	34.02467	0	300
Hospital	226	-	-	-	-
Treatment group					
Total spent	486	15.85401	37.97682	0.1	665
Gender	485	0.5484536	0.4981605	0	1
Age	485	50.96701	15.36804	8	92
Insurance status	486	0.8312757	0.3748941	0	1
Education	486	2.026749	0.5791075	1	4
Illness	486	2.925926	0.7054038	1	4
Income	486	46.82597	45.80607	0	550
Hospital	426	-	-	-	-

It can be seen that the treatment group paid on average one third of the control group’s total spending. The gender decomposition within the control group and the treatment group is roughly equal. However, there are marked differences between the control and treatment groups in terms of age, insurance status, and income distributions. A two-sided *t*-test both in the simple regression equation and in the model that controls for the X covariates reveals that the impact of the treatment indicator on the outcome variable is negative and statistically significant at any standard level of significance. While this gives some hints about the effects of the explanatory variables on healthcare costs along with the direction of the impacts, the results provided by ordinary least squares estimations are likely to be biased due to the presence of confounders. In particular, while control and treatment subjects have, on average, similar degrees of illness severity (Bad) and highest levels of education (High School) and similar deviations from the reported means of these variables, there are stark differences in terms of age, insurance status, and income. The control group is expected to be poorer, younger, and less likely to be insured than the treatment group. Prior to comparing the outcomes, therefore, it is necessary to match on the observed characteristics to ensure that the observations are moderately “similar”.

### Propensity score matching

Following the algorithm detailed in section "Methodological framework", propensity scores are estimated prior to stratification matching. The average treatment effect is then calculated. In the final analysis, two alternative specifications are employed to match propensity scores between treated units and control units to ensure the robustness of the baseline model.

#### Checking the balance of confounders between treated and untreated units

The earlier descriptive statistics hints at a potential mismatch in the observed covariates between the treatment and control groups. Nevertheless, as these variables are measured in different units, it is not clear which difference is more important than the others. A possible approach to make this distinction is to conduct significance tests, but this approach is prone to two main issues within the present context: first, it is highly sensitive to sample size, and second, it fails to provide information the magnitude of the differences, if any. This necessitates examining the differences in terms of standard deviations. Table 3 shows pre-matching results on the observed covariates between the treatment and control groups.

**Table 3:** Pre-matching analysis of the observed covariates

	Mean in treated	Mean in control	Standardized difference
Gender	0.53	0.6	-0.148
Age	52.01	36.7	0.909
Insurance status	0.9	0.49	0.987
Education	1.99	2.11	-0.208
Illness	2.95	3.21	-0.366
Income	44.02	36.27	0.224
Hospital	77.17	132.47	-1.547

It is clear from the table that there exist significant imbalances in most of the control variables, with the borderline exceptions of gender, education, and income level. This agrees with our descriptive analysis presented above and shows that the treatment and control groups are more similar with respect to these variables than to the remaining controls. It should be noted that the reported means for *Hospital* do not have any realistic meaning; the variable is encoded to facilitate subsequent matching.

#### Estimating propensity scores

**Fig. 1:**
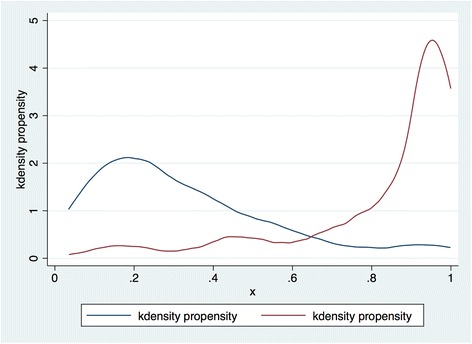
Propensity score distributions

**Fig. 2:**
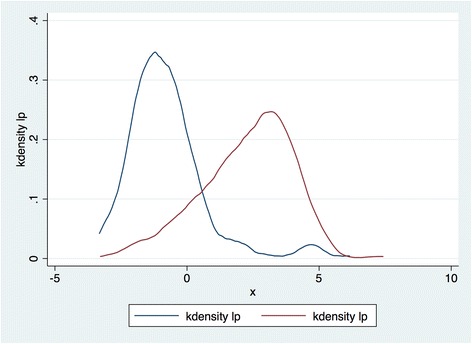
Linear predictor distributions

Logistic regression is used to estimate the propensity scores. Given the categorical nature of most of the control variables, matching is conducted with distinct categories in predicting the propensity scores. The distributions of propensity scores within the treatment and control groups are shown in Fig. 1. Given the upper and lower bounds on propensity scores, both distributions are skewed. As a result, we would use the log of the odds of the predicted propensity score (referred to as the “linear predictor” henceforth), rather than the propensity score itself. With this non-linear transformation, the distributions have become much more normal in both of the subgroups, as shown in Fig. 2.

#### Stratifying the estimated linear predictors

Once the transformation on the predicted propensity scores has been made, our subjects are stratified into 5 levels. Table 4 shows quantiles of the estimated log odds. It can be seen that every quantile contains at least some members of the treatment and control groups, satisfying the common support assumption and rendering it possible to calculate the average treatment effect within each stratum. Within the lowest quantile, over three fourths of the subjects do not receive the treatment, whilst in the highest quantile, almost all subjects receive the treatment.

**Table 4:** Five quantiles of the linear predictor

Quantile	Residency = 0	Residency = 1	Total
1	110	20	130
%	84.62	15.38	100
2	85	45	130
%	65.38	34.62	100
3	21	109	130
%	16.15	83.85	100
4	3	127	130
%	2.31	97.69	100
5	7	123	130
%	5.38	94.62	100

#### Rechecking covariate balance

The next step is to check the balance after stratification. This step is crucial, as it would give necessary evidence to support causality. Table 5 shows post-matching results for both groups. It can be seen that after stratified matching based on the linear predictor, all the standardized differences have shrunk considerably toward zero, allowing us to safely conclude that the matching process has been successful.

**Table 5:** Checking balance of confounders after stratification

	Mean in treated	Mean in control	Standardized difference
Gender	0.53	0.52	0.026
Age	52.01	51.09	0.055
Insurance status	0.9	0.9	-0.009
Education	1.99	1.99	0
Illness	2.95	2.96	-0.02
Income	44.02	43.36	0.019
Hospital	77.17	78.66	-0.042

#### Average treatment effect assessment

The final step involves assessing the average treatment effect across strata of the linear predictor. A pooled, naive regression yields an estimated treatment effect of -27.87 (SE = 2.78). While this result is highly statistically significant, it is biased due to the presence of the confounders discussed above. Therefore, in order to evaluate the treatment effect more accurately, a binary indicator that represents each of the strata to the regression equation is added. The remedial estimation results are shown in Table 6. It can be seen that the estimated average treatment effect is approximately -15, and is statistically significant at α =0.01. The 95% confidence interval also confirms the negative sign of the treatment effect in our initial hypothesis. Not surprisingly, the average treatment effect has declined in magnitude once the presence of confounders is taken into account.

**Table 6:** Average treatment effect for 5 strata of the linear predictor

Total spending	Coefficient	Std. Err.	t	*P*-value	95% CI	
Residency	-14.11	2.95	-4.79	0.00	-19.90	-8.32
Quantile 2	-0.03	3.19	-0.01	0.99	-6.28	6.23
Quantile 3	-15.59	3.73	-4.18	0.00	-22.91	-8.27
Quantile 4	-18.83	3.96	-4.75	0.00	-26.61	-11.05
Quantile 5	-16.44	3.91	-4.21	0.00	-24.11	-8.76
Constant	40.24	2.26	17.78	0.00	35.79	44.68

#### Robustness checks

This section presents two methods used to check the robustness of the proposed model under alternative specifications.

##### Decile stratification

It is conventional wisdom that 5 strata of the propensity scores (or in this case, linear predictors) are expected to remove confounding by as much as 90%. We will test the robustness of our previously-obtained results by stratifying the estimated linear predictors into finer groups. The results show that 10-level stratification gives finer and better covariate balancing, though the average treatment effect is almost the same as one obtained in the original specification in both sign and magnitude.

##### Reweighting the log odds ratio

The second alternative specification follows Sato and Matsuyama [19] and involves reweighting the data to equalize confounders across the treatment and control groups. In other words, reweighting is employed to remove the effect of confounders in estimating the average treatment. Within our context, standardized morbidity ratio (SMR) weights are used to eliminate the association between the treatment and confounders. Table 7 shows that once SMR standardization is applied, the average treatment effect is estimated at approximately -13.89, which is rather close to the result obtained from the baseline model.

**Table 7:** Alternative specifications of the baseline model

Alternative specification	ATE	Std. Err.	t	*P* > t	[95% Conf.	Interval]
Decile stratification	-14.55453	3.017136	-4.82	0.00	-20.47923	-8.629827
SMR reweighting	-13.88859	7.825919	-1.77	0.077	-29.26865	1.491484

Overall the results are highly consistent and statistically significant across different specifications, showing that the estimated treatment effect is highly robust.

### Sources of the inequality in healthcare costs

The above findings give evidence to conclude that patients who reside in the same regions in which the hospitals are located have an average reduction of approximately 15 million VND (around 750 USD) in total healthcare costs, compared to those who are non-residents. This inequality is sizable, given the distribution of total healthcare costs for the overall sample.

In order to gain deeper insights into the mechanism of how the differences in residency status create such considerable inequality in total healthcare spending, we start with Vuong & Nguyen [13]’s insightful observation that patients from a different region than the region where the hospital is located typically spend a significant amount of money supporting their accompanying relatives during hospitalization. To extend Vuong & Nguyen’s insights even further, we also relate residency status to the number of days spent in the hospitals. The uniqueness of the dataset associated with this study also allows testing the hypothesis that residency status determines the amount of “courtesy money,” a form of covert bribery that patients typically give doctors in Vietnamese hospitals in order to receive preferential treatment. Multivariate linear regressions are employed to quantify these predictions; the results are given in Table 8.

**Table 8:** Parameter estimates for multiple linear regressions

Dependent Variable	Spending on Relatives	Courtesy Money	Days in Hospital
Residency status	-1.36^a^	-0.60^a^	-2.30^a^
	(0.30)	(0.19)	(0.43)
Gender	-0.04^a^	-0.04	0.01
	(0.27)	(0.17)	(0.39)
Age	-0.03^a^	0.00	-0.01
	(0.01)	(0.01)	(0.01)
Insurance coverage	0.26	-0.61^a^	1.64^a^
	(0.32)	(0.20)	(0.45)
Education	-0.32	0.93^a^	-0.66^b^
	(0.23)	(0.15)	(0.34)
Illness	0.83^a^	0.40^a^	2.41^a^
	(0.19)	(0.12)	(0.27)
Constant	2.04^a^	-1.26^b^	3.87^a^
	(0.90)	(0.56)	(1.29)

Standard errors are in parentheses. ^a^ and ^b^ stand for levels of significance at 0.01 and 0.05, respectively

The regression analyses indicate the existence of three potential sources for the observed total healthcare spending differences. The individual significance and negative sign of the variable *Residency status* across the three panels allow retaining the hypothesis that there is a statistically significant (α =0.01) difference in spending on accompanying relatives, courtesy money, and days of hospitalization between residing and non-residing patients.

To be specific, if a patient resides in a different region from the location of his or her treatment hospital, the patient is expected to pay about $1.36 million VND (approximately $55 USD) higher for the relatives who accompany him or her to the hospital than residing patients do. This gives a solid justification for our main result that there is a significant gap between residing and non-residing patients’ total healthcare costs.

Understandably, it takes an average non-residing patient 2–3 days more to spend in hospitals than it does an average residing patient. This might have to do with the paperwork and bureaucratic procedures which might vary from patient to patient, depending on their residency locations.

The most interesting result from our regression analysis is that there exists a statistically significant difference between residing and non-residing patients in terms of the amounts designated for “courtesy funds.” To be specific, a residing patient pays 600 VND (or about 30 USD) lower in courtesy money than a non-residing patient, on average.

## Discussion

###  Limitations of the study

The present analysis suffers several limitations, which at the same time would provide various opportunities for further explorations and extensions. The first limitation concerns data collection and processing. While Vuong & Nguyen’s effort in making the sample as representative of the Vietnamese patient population as possible is commendable, common issues regarding non-probability sampling are inevitable. The lack of control for healthcare services is another drawback of the study. While medical institutions are controlled for in estimating and matching the propensity scores, the specific breakdown of healthcare services utilized by the hospitalized patients would provide much more comprehensive information for matching. For example, patients treated at specialist hospitals might request different sets of services from those offered at general hospitals, associated with which there are different costs. This consideration is particularly relevant to the study, as the variation in service costs is expected to contribute to inequality in total healthcare spending. Uncontrolled for, these services would likely confound the findings.

The final limitation is related to the study’s methodology. While our matching strategy here addresses some of the methodological drawbacks in the previous literature, it is not without fault. In particular, the propensity score matching framework employed in this paper only takes into account observed covariates. There may exist potential unobservables that should also be accounted for in matching. In this regard, a structural equation modelling framework could provide a more complete picture and perhaps allow the researcher to answer more interesting questions.

### Policy recommendations

A possible explanation for the remarkable reported gap in spending between the residing and non-residing groups stems from a unique prevalent practice in the Vietnamese society, “*Mot con ngua dau ca tau bo co*,” literally translated as “When one horse is sick, the whole herd refuses to continue eating,” which in this case highlights the fact that whenever a family member is hospitalized, the whole family will stop their business temporarily, go to the hospital, and literally stay there to take care of the patient. This practice imposes additional financial constraints on the total healthcare costs that each patient incurs, especially for non-residing patients.

Also seen from the regression analysis is the interesting result that there exists a statistically significant difference of about 30 USD between residing and non-residing patients in “courtesy funds.” This amount is *not* negligible, given that a typical worker living in the capital of Vietnam earns only 145 USD per month, according to recent estimates for 2015. Our result supports the hypothesis that there is some degree of inequality facing non-residents in bribery practices in Vietnamese hospitals, placing additional burdens on this group of patients.

## Conclusionss

Despite the above apparent limitations, this study makes several contributions to the literature. First, it gives preliminary causal evidence for the hypothesis that differences in residency status lead to inequality in total healthcare costs. This result adds an interesting dimension to previous healthcare spending literatures and signals to policymakers and hospital administrators the potential sources for healthcare spending discrepancies as well as the possible ways to improve accessibility for patients.

The regression analysis identifies the three main contributors to the differences in residency status – namely spending on relatives, courtesy funds, and days of hospitalization. While the first of these factors is driven primarily by individual decisions, the remaining two concern more with institutional characteristics, suggesting a potential arena for policymakers to narrow the spending gap between residing and non-residing patients in Vietnamese hospitals. In particular, from our study results, it might be tempting to suggest that efforts should be made to reduce the number of days spent in hospitalization. This would potentially help alleviate the cost burdens for non-residing patients and reduce efficiency loss from rationing. However, this recommendation warrants some valid skepticism, as reducing the number of hospitalized days without taking into account the heterogeneity in health conditions might be detrimental to patient health. As a result, differences in specific health services as well as medical conditions also ought to be considered before such a general recommendation can be made.

An interesting result from the study is that non-residing patients in Vietnam have to spend more than residing patients do on bribing hospital doctors, on top of the actual treatment costs that both groups are entitled to pay. To the extent that this study can be generalized, it appears that medical institutions can partially address inequality in healthcare spending with more effective policing of anti-corruption policies.

Given the limitations discussed above, there are a number of ways in which the present study can be extended. First, most of the analysis in this paper deals only with explicit costs to the patients. However, as mentioned earlier, since hospitalization in Vietnam typically involves not only the patients themselves but their relatives as well. Future studies could illuminate the present discussion further by taking into account and quantifying the various types of opportunity costs associated with this phenomenon. In addition, the costs of accompanying relatives are related to the residency statuses of these relatives themselves. Thus, future studies could investigate whether the reported gap between residing and non-residing patient groups can be classified as inequality and can provide meaningful policy implications. In terms of methodology, the flexibility of the propensity score matching procedure employed in this paper allows this method to be applicable to various other contexts. Future studies could, for instance, investigate the causal impact of other variables, including patients’ occupation, number of family members, among others, on total healthcare spending. This would further deepen the insights that the previous literature has provided.

## Abbreviations

ATE: Average treatment effect
GDP: Gross domestic product
SMR: Standardized morbidity ratio
USD: US dollar (United States’ currency)
VND: Vietnamese dong (Vietnamese currency)
